# Ocean Warming–Acidification Synergism Undermines Dissolved Organic Matter Assembly

**DOI:** 10.1371/journal.pone.0118300

**Published:** 2015-02-25

**Authors:** Chi-Shuo Chen, Jesse M. Anaya, Eric Y-T Chen, Erik Farr, Wei-Chun Chin

**Affiliations:** 1 School of Engineering, University of California Merced, Merced, California, United States of America; 2 School of Natural Sciences, University of California Merced, Merced, California, United States of America; Auckland University of Technology, NEW ZEALAND

## Abstract

Understanding the influence of synergisms on natural processes is a critical step toward determining the full-extent of anthropogenic stressors. As carbon emissions continue unabated, two major stressors—warming and acidification—threaten marine systems on several scales. Here, we report that a moderate temperature increase (from 30^°^C to 32^°^C) is sufficient to slow— even hinder—the ability of dissolved organic matter, a major carbon pool, to self-assemble to form marine microgels, which contribute to the particulate organic matter pool. Moreover, acidification lowers the temperature threshold at which we observe our results. These findings carry implications for the marine carbon cycle, as self-assembled marine microgels generate an estimated global seawater budget of ~10^16^ g C. We used laser scattering spectroscopy to test the influence of temperature and pH on spontaneous marine gel assembly. The results of independent experiments revealed that at a particular point, both pH and temperature block microgel formation (32^°^C, pH 8.2), and disperse existing gels (35^°^C). We then tested the hypothesis that temperature and pH have a synergistic influence on marine gel dispersion. We found that the dispersion temperature decreases concurrently with pH: from 32^°^C at pH 8.2, to 28^°^C at pH 7.5. If our laboratory observations can be extrapolated to complex marine environments, our results suggest that a warming–acidification synergism can decrease carbon and nutrient fluxes, disturbing marine trophic and trace element cycles, at rates faster than projected.

## Introduction

Existing as part of an organic matter continuum, the ability of dissolved organic matter (DOM) polymers to spontaneously assemble into a more bioactive microgels represents a 70 Gt carbon flux [1,2], out of a total DOM budget of 700 Gt carbon. The DOM–particulate organic matter (POM) shunt plays many roles: it redirects organic carbon flow in marine microbial communities [3–5]; reshapes trophic cycling [6–8]; and even serves as cloud condensation nuclei [9]. Conventionally, DOM has been considered a refractory macromolecule, revealing complex chemical compositions and structures [1,10]. However, notwithstanding their broad significance, it remains unknown whether the macromolecular nature of DOM is susceptible to multiple environmental fluctuations—fluctuations realistic under future climate scenarios [11,12]. Considering the critical nature of the DOM–POM shunt, minute perturbations to DOM assembly, induced by moderate temperature or pH changes, would have effects on the ocean carbon flux and marine ecosystems [13].

## Materials and Methods

### Experimental Design

We conducted five independent experiments to investigate the influence of temperature and pH on marine gel assembly. Our hypotheses were: 1) there is a particular point for both temperature and pH beyond which marine gels cannot assemble and existing gels are dispersed; and 2) there is a warming–acidification synergism to microgel assembly. The first experiment investigated an upper bound for temperatures beyond which marine gels could not assemble. The second experiment investigated the potential dispersion of microgels to temperatures above 30°C. The third experiment investigated an upper bound for pH beyond which marine gels could not assemble. The fourth experiment investigated a potential warming–acidification synergism on an upper bound beyond which marine gels could not assemble. The fifth experiment evaluated Ca^2+^, a DOM polymer cross-linker, and marine gel hydrophobicity, an additional DOM polymer cross-linking mechanism [1,14], to assess which mechanism might contribute to the assembly/dispersion. All data are reported with the mean and standard deviation.

### Water Sampling and Filtration

Seawater samples collected at Puget Sound (WA, USA) near Friday Harbor Marine Laboratories in August 2009 were filtered through a 0.22-µm membrane (Millipore polyvinylidene fluoride low protein binding filter, prewashed with 0.1 N HCl), treated with 0.02% sodium azide—a microbial biocide—and stored in sterile, Parafilm-sealed bottles in the dark at 4°C until further processing. Specific permission was not required to collect seawater sample (<5 L) at this site (Friday Harbor, WA, USA, 48.54619, -123.00761). No endangered or protected species were involved. DOC (dissolved organic carbon) concentration (2.566 mg L^-1^) was measured using a Shimadzu TOC-Vcsh Total Organic Carbon Analyzer [15].

### Growth Kinetics

Microgel assembly was monitored with dynamic laser scattering as described previously [4]. Seawater aliquots (10 mL) were syringe-filtered through a 0.22-µm membrane (low-protein binding Durapore, Millipore) directly into scintillation vials (pre-rinsed with Milli-Q Millipore DI water). Scattering cells were placed in the goniometer of a Brookhaven laser spectrometer (Brookhaven Instruments, NY), where the scattering fluctuation signals were detected at a 45° angle. The autocorrelation function of scattering intensity fluctuations was averaged over a 12-minute sampling time. Hydrodynamic diameters of polymer gels were analyzed by the CONTIN method [1,4,16]. Seawater samples were measured every 24 or 72 hrs by dynamic laser scattering to monitor size changes.

### Temperature-pH Adjustments

**Fig 1:**
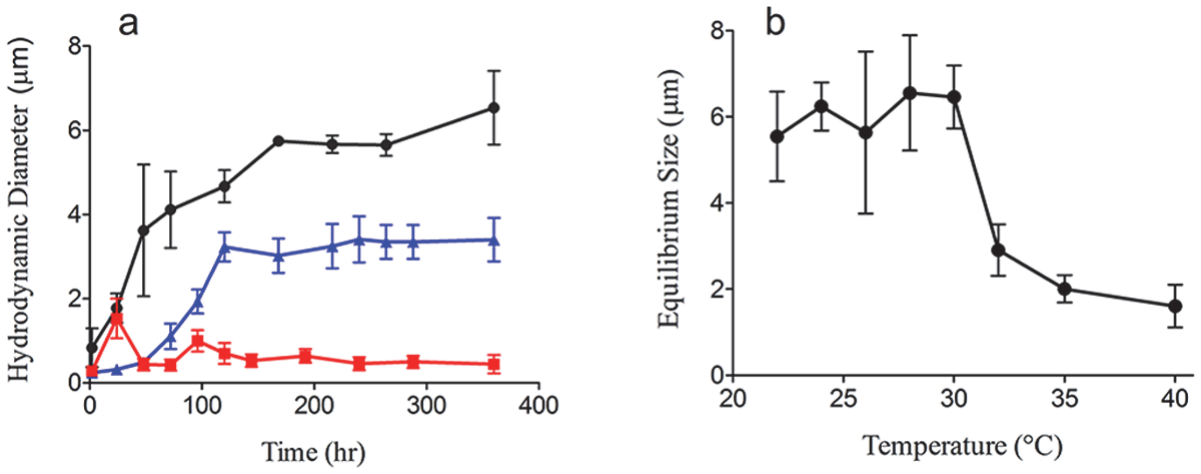
Microgel assembly / dispersion are temperature dependent. 1-a. Microgel assembly rate and equilibrium size decreases with increased temperature. Samples were incubated at 22˚C (black circles), 32˚C (blue triangles) and 35˚C (red squares) for 24 hours, then stored in the dark at 22˚C for the remainder of the experiment. Assembly was measured using dynamic laser scattering at 22˚C. Each data point represents (mean ± SD) of six measurements made in each of six replicate samples. Data highlight that short-term temperature exposure above 35˚C confers significant DOM assembly loss with no obvious recovery. 1-b. Microgel dispersion depends on temperature variation. Self-assembled microgels (size ~ 6 μm) were incubated at various temperatures (from 22˚C to 40 ˚C) for 24 hours. The equilibrium microgel sizes were monitored with dynamic laser scattering spectroscopy. Each data point represents six replicate samples. Non-linear temperature responses of microgels were observed—particularly for microgels incubated at temperatures above 32˚C, which showed a marked size decrease.

**Fig 2:**
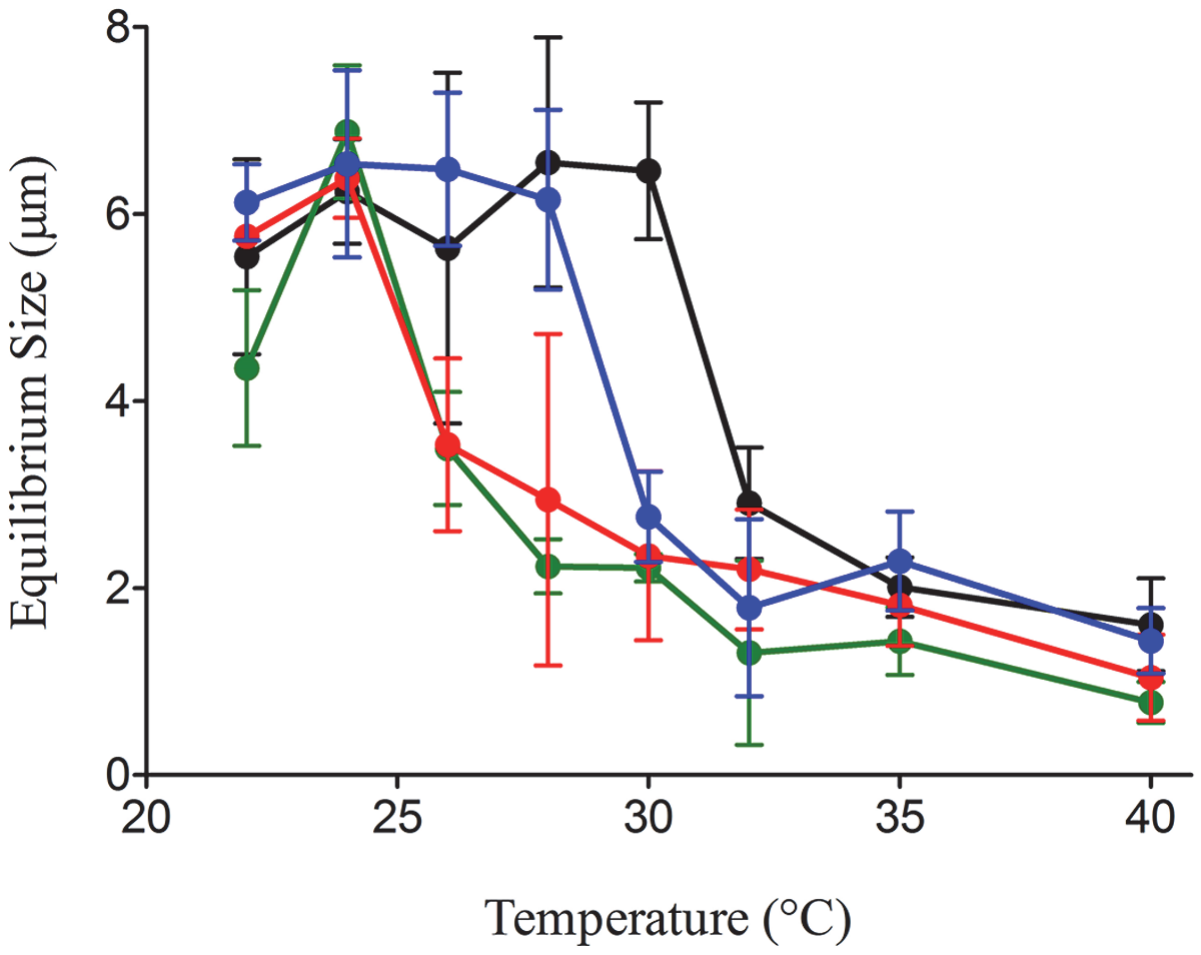
Dispersion-temperature of microgels decreases at lower pHs. As pH is reduced from 8.0 (black) to 7.7 (blue) to 7.5 (red) to 7.3 (green), non-linear microgel dispersion-temperature changes were observed while pH decreases. Dispersion temperature dropped ~2˚C with a 0.2 pH decrease. Each data point represents mean (+/−SD) of six measurements made in each of six replicate samples.

**Fig 3:**
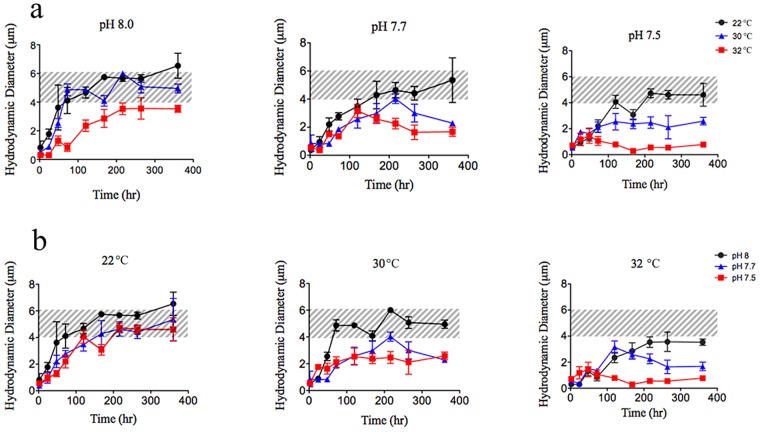
DOM assembly monitored with temperature and pH reveals that, as either pH decreases or temperature increases, microgel equilibrium size and assembly rates decrease at a non-linear rate. a. DOM assembly at three temperatures—22˚C (black circles), 30˚C (blue triangles), 32˚C (red squares)—over time at three pH units. Each data point represents (mean ± SD) of six measurements made in each of six replicate samples. b. DOM assembly at three pHs—8.0 (black circles), 7.7 (blue triangles), 7.5 (red squares)—over time at three constant temperature incubations. Microgels assembled in identical pH conditions showed equilibrium size reduction and decelerated non-linear assembly rates when exposed to increased temperature. Each data point represents the mean (+/− SD) of six measurements made in each of six replicate samples. Shaded windows represent an average microgel equilibrium size range (4–6 μm) at 22˚C and pH 8.

Based on climate changes models [17,18], we set up the ranges of experimental temperature and pH. We understand only specific regions may experience wide-range temperature/pH changes; however, we aimed to explore potential impacts and possible consequences under extreme environmental conditions. In temperature-dependent experiments (Fig. 1), samples in airtight, sealed scintillation vials were grouped and incubated at 22, 32 and 35°C for 24 hrs and stored/monitored for 15 d at 22°C. For microgel dispersion-temperature experiments (Fig. 2), 10 mL of seawater were syringe-filtered into vials and incubated in dark for 10 d at 22°C. After confirming microgel equilibrium sizes by dynamic laser scattering, seawater sample pHs were adjusted with 0.1N HCl without an extra buffer system, and seawater samples were incubated at desired experimental temperatures for 24 hrs. Microgel sizes were measured with dynamic laser scattering spectroscopy immediately after pH/temperature adjustments. For the combined pH-temperature impact on microgels experiment, DOM assembly was monitored under pHs ranging from 7.3 to 8.0 and temperatures ranging from 22 to 32°C (Figs. 2 and 3). 0.1N HCl was used to adjust seawater samples to desired pH, and scintillation vials were incubated and measured at each experimental temperature over 15 d.

## Results and Discussion

### Increased Temperature Hinders DOM-Microgel Assembly

One of the most salient outcomes of climate change is surface warming, projected to increase by 3°C, relative to the 1961–1990 mean, in certain ocean regions by the end of the century [19]. These projections involve temperatures above 30°C [14,17], a previously studied temperature limit. To investigate increasing temperature effects on DOM, we monitored microgel assembly as a function of time using dynamic laser scattering spectroscopy as described previously [4]. DOM in seawater (Friday Harbor, WA, USA), passed through a 0.22 μm-filter, was incubated (at 22, 32 and 35°C) for 24 hrs before assembly was monitored. Our control group (22°C) showed DOM polymers can spontaneously assemble into gels with sizes ranging from 200 nm to 1 μm within 30 minutes. Microgels continued to grow following a nonlinear assembly process to reach equilibrium sizes (~5 μm) within 100 hrs. Unexpectedly, microgel assembly was hindered after 24-hr incubation at 35°C (Fig. 1a). Our results show that increasing seawater temperature progressively hinders microgel assembly. DOM exposed to 35°C can undergo long lasting inhibition of multi-micrometer gel (microgel) formation, yielding only nanogels of less than a micrometer in dimension. No subsequent micron-scale assembly following initial dispersion was observed after 10 days of monitoring.

DOM polymers can spontaneously assemble to form microgels [11]. In order to further test the impacts of increased temperature on DOM self-assembly, we performed another experiment (Fig. 1b). We measured DOM that had reached its equilibrium size (120 hrs at 22°C), first confirming the equilibrium size of microgel (~6 µm) using dynamic laser scattering spectroscopy. Our results from dynamic laser scattering showed that microgel sizes reduce considerably—from 6 µm to 2 µm—after incubation at temperatures above 32°C for 24 hrs (Fig. 1b). Contrary to the previously reported reversible volume transition through pH/temperature titration within a short exposure time [4,11], the significant microgel size reduction observed here appears irreversible (Fig. 1a). The instability (size changes) of DOM microgels above 32°C is consistently evident in Figs 1a and 1b, implying potential impacts of higher temperatures on organic matter.

The non-linear temperature dependency demonstrates one of the polymer characters of microgel kinetics—but implies even short-term environmental perturbation may cause long-term impacts. If our experimental findings in the lab can be extrapolated to complex marine environments, our results indicate that increasing temperatures may impede DOM’s ability to form microgels, thereby potentially disrupting the marine colloidal pump—a major carbon cycling driving mechanism, and leading to decreasing downward carbon flux from the surface ocean to the deep ocean [11,20].

### Possible Mechanism for Micro/Nano Marine Gel Transition

**Fig 4:**
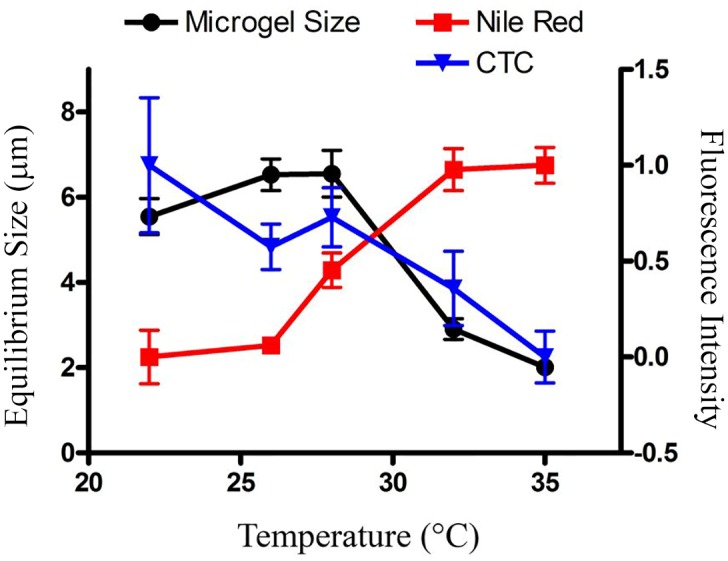
Decreased microgel equilibrium size (black circles) and bound Ca^2+^ (blue triangles) with concomitant increase in hydrophobicity (red squares). The non-linear rate of declining microgel size with increased temperature indicates potential cooperativity; around 32˚C all three parameters experienced the most pronounced associative effect—a major drop in microgel size and bound Ca^2+^, with a concomitant rise in hydrophobicity.

To probe the mechanism of gel assembly changes, we studied microgels using spectrofluorophotometry. Two major mechanisms of DOM cross-linking have been demonstrated: Ca^2+^ cross-linking and hydrophobic binding [1]. Divalent ion (Ca^2+^) cross-linking has been identified as a major driving mechanism for DOM microgel formation [1,4]. We used chlortetracycline (CTC) to quantify bound Ca^2+^ on DOM polymers to assess the relative contribution of Ca^2+^ cross-linking [4,21,22]. The CTC fluorescence intensity of heated DOM decreased ~50%, which indicated fewer bound Ca^2+^ ions on polymer surfaces (Fig. 4). Thus, decreased levels of bound Ca^2+^ on DOM polymers associated with increased temperatures would explain smaller equilibrium sizes of forming gels.

Additionally, DOM can be cross-linked by changes in hydrophobic binding [1]. We used Nile red staining to evaluate relative hydrophilic/hydrophobic changes for DOM polymers [23]. Compared to native DOM—with low hydrophobicity (Fig. 4)—our Nile red data revealed increased hydrophobic ratios on heated DOM polymers.

Increasing hydrophobicity leads to an increase in DOM polymer association [14], either via intermolecular or intramolecular interactions. Within the moderate temperature range studied here, the observed nanogel formation (microgel size decrease), DOM Ca^2+^ binding and associated hydrophobic/hydrophilic differences strongly suggest involvement of polymer conformational changes. We speculate different polymer conformations favor the stable nanogel formation, rather than polymer chain entangling, which serves as a major driving force for the observed gel size decrease. From the perspective of polymer assembly, in the absence of covalent cross-linkers, an increase of polymer reptation concomitants a decrease of entangle frictions [24]. In contrast to extended polyionic coils, polymer globule conformation resulting from increased hydrophobicity decreases the intermolecular polymer tangle interactions, which lead to the change of assembly dynamics and final gel equilibrium size. Due to abundant anionic charges and functional groups on DOM polymers, as H^+^ concentration increases (owing to a pH decrease), H^+^ ions can protonate certain functional groups on DOM polymers, causing fewer available binding sites for divalent Ca^2+^ cross-linking [1,4]; fewer cross-linked polymer chains would result in longer correlation lengths and decreased entangle friction [24]. Thus, low pH situations would lead to smaller equilibrium microgel sizes, as observed in our results (Fig. 3). To verify the influences of low pHs on the stability of microgel matrices, we monitored the impacts of temperatures on microgels at different pHs. Given fewer available cross-linking sites, as hypothesized, the critical temperature decreased with lower pHs: to 30°C at pH 7.7 and again to 28°C at pH 7.5 (Fig. 3). The model of polymer conformation was supported by our current results. However, without direct DOM conformation measurement, future investigation will be needed to further verify this proposed model.

### Microgel/Nanogel Transition Temperature Lowered with Increased Acidification

Microgel assembly is dynamic, stable and reversible in environments up to 30°C [11]. However, because recent ocean warming trends and climate model predictions point to temperatures above this threshold [17,18], we investigated microgel stability in higher temperature environments. In addition to warming, ocean pH is projected to decrease by as much as 0.5 pH units by 2300 [25,26]. To accommodate the more realistic scenario of simultaneous temperature and pH fluctuations, DOM assembly was investigated at 3 pH values (7.5, 7.7, 8.0) coupled with 3 temperature conditions (22, 30, 32°C). We found the equilibrium microgel size decreased with increased temperature, although microgel size at pH 8.0 remained ~4 μm, even at 32°C (Fig. 3). Dropping pH from 8.0 to 7.5 at 22°C, DOM assembly rates decreased (from 120 to 200 hrs), as well as equilibrium sizes (from ~6 to ~4 μm). At pH 7.5, for temperature >30°C, microgel equilibrium sizes dramatically decreased to <2 μm. In addition, the transition temperature of microgels to nanogels was investigated under different pHs (Fig. 2). In general, we found temperature impacts on DOM assembly were amplified with a concurrent pH decrease: at pH 7.5, the microgel size reached only ~0.5 μm at 32°C. This relationship highlights an unexpected synergism—i.e., increasing temperature and acidification—on microgel formation to disturb DOM-microgel exchange and potentially impact the marine carbon cycle.

### Potential Far-Reaching Influences on Marine Processes

Our results that demonstrate DOM susceptibility to moderate environmental changes provide alternative scenarios for DOM utilization by microbial communities. Microbial respiration depends on the bioavailability of organic carbon in the water column, which is lower for DOM than for microgels [3,27]. In addition, several mechanistic pathways have also been proposed for refractory DOM formation; these include ectoenzyme conversion and exudation during bacterial production [28] as well as hydrophobicity and micelle-like macromolecular structures—proposed to contribute to DOM degradation resistance [29]. The hydrophobicity increase induced by environmental changes observed here permits an understanding of an alternative abiotic mechanism for refractory DOM formation.

The ocean colloidal pump plays an important role in the carbon cycle [30], downward nutrient transport, and organic particle dynamics in the ocean [1,11]. It also has been reported that small size particles (<10 µm) play an important role for carrying organic carbon out of the euphotic zone [31]. The sensitivity of the DOM/microgels shunt to temperature and pH fluctuations reveals the vulnerability of this critical driving force (i.e., the colloidal pump) in future climate change scenarios. Ocean acidification changes the algal metabolism [32]; one mesocosm study showed warming can increase the extracellular release of organic materials from phytoplankton [33]. Environmental impacts resulting from abiotic micro/nanogel transitions warrant further investigations to better understand this complicated system. First, a possible reduction of downward organic carbon fluxes driven by the colloidal pump may be expected. The decreased burial of organic carbon on the seafloor could dampen the capacity of the oceanic carbon sink. In addition, a decrease in microgel downward flux can partially reduce nutrient transport that sustains microbial communities in the twilight (200–1000 m) and deep-sea (>1000 m) regions [5,6,34]. As self-assembled gels are found over a broad range, from the surface to 4000 m depth, this shift of organic carbon supply to the microbial communities would stress biodiversity in the deeper oceans [35], though the magnitude of this disturbance—or any other—remains uncertain. In the surface ocean, reduced microgel assembly could restructure nutrition availability for colonized bacterial communities [1,11,36]. Furthermore, it has been shown that DOM assembly may affect trace metal cycling and the marine biological pump [1], which mediates carbon fixation through the photosynthesis of surface ocean phytoplankton [6,34]. The observed micro/nanogels transition not only indicates the changes of surface area/volume ratio of marine gels, but the different surface properties observed in our study. Studies of the interaction between trace elements and nanogels will significantly contribute to our understanding of the capacity of biological carbon pump in future warmer, more acidic ocean environments [26].

Assessing how climate changes will affect global carbon cycling is one of Earth sciences’ great challenges. Our findings suggest ocean acidification and warming, both the result of unabated CO_2_ levels, would significantly, and irreversibly, alter marine DOM and DOM/microgel dynamics. These effects reshape the picture of DOM as chemically stable and refractory. Instead, they reveal a more nuanced view of the DOM/microgel transition—one that reveals a critical point sensitive to pH and temperature. From the surface ocean to the ‘dark side’—depths past the euphotic zone, through DOM and its associations within the carbon cycle—we propose this synergism of ocean warming and acidification carries potential to be far-reaching, likely affecting major carbon fluxes and microbial communities. Our findings imply a novel potential synergism from seemingly independent changes (e.g., increased temperature and ocean acidification) may hold significant impacts on carbon cycle dynamics and stress the urgency to study marine processes as parts of an integrated system.
